# PB.17: Inter-observer agreement in visual analogue scale assessment of percentage breast density

**DOI:** 10.1186/bcr3517

**Published:** 2013-11-08

**Authors:** JC Sergeant, M Wilson, N Barr, U Beetles, C Boggis, S Bundred, M Bydder, S Gadde, E Hurley, A Jain, Y Lim, L Lord, V Reece, DG Evans, A Howell, SM Astley

**Affiliations:** 1 Centre for Imaging Sciences, Institute of Population Health, University of Manchester, UK; 2 Nightingale Centre and Genesis Prevention Centre, University Hospital of South Manchester, UK

## Introduction

Breast density is an important risk factor for breast cancer. Assessment of density at screening could help identify women at increased risk of cancer, who may benefit from screening with shorter intervals or different modalities. Visual analogue scale (VAS) assessment of percentage density by observers is straightforward to implement and strongly associated with cancer risk. However, using VAS assessment for stratification would require reproducibility between observers. We examine agreement between observers assessing VAS density.

## Methods

The VAS breast density of 120 screening cases with full-field digital mammograms was independently assessed by 12 experienced mammographic readers. The agreement between the readers was assessed using Bland-Altman limits of agreement and the concordance correlation coefficient (CCC). The VAS densities were also converted to BIRADS breast composition categories and agreement measured with Cohen's weighted kappa.

## Results

The greatest difference between two estimates for the same case was 67.75 percentage points, while the mean difference between reader pairs ranged from 0.76 to 28.58 percentage points. The 95% limits of agreement between reader pairs were (-6.96, 18.62) at their narrowest and (-59.13, 1.97) at their widest. Pairwise CCC values ranged from 0.44 to 0.92, while the overall CCC was 0.70. Pairwise kappa values for the BIRADS classification ranged from 0.37 to 0.84, with a mean of 0.66.

## Conclusion

Substantial lack of agreement was found between readers visually assessing percentage breast density. This study demonstrates the need for reader harmonisation, either by training or by adjustment of results, should VAS density assessment be used for risk stratification.

